# Interdisciplinary medication reviews of psychiatric patients – A mixed method evaluation

**DOI:** 10.1016/j.rcsop.2025.100584

**Published:** 2025-03-02

**Authors:** Dagmar Abelone Dalin, Sara Sommer Holst, Lucif Søemosegaard Dalin, Charlotte Vermehren

**Affiliations:** a Department of Clinical Pharmacology, Copenhagen University Hospital Bispebjerg, 2400 Copenhagen, Denmark; b Capital Region Pharmacy, Copenhagen University Hospital Bispebjerg, 2400 Copenhagen, Denmark; c Department of Drug Design and Pharmacology, Faculty of Health and Medical Sciences, University of Copenhagen, 2200 Copenhagen, Denmark

**Keywords:** Medication review, Psychiatry, General practice, Pharmacist, Interdisciplinary team, Polypharmacy

## Abstract

**Background:**

Patients at psychiatric homes are a vulnerable group with several factors complicating their pharmacological treatment. Psychiatric patients in stable treatment are transferred from specialist care to primary care, which presents new challenges as general practitioners (GPs) may feel that they are not sufficiently trained in prescribing and tapering psychiatric drugs. Medication reviews (MRs) have been seen to improve the appropriateness of pharmacological treatment – especially when performed in interdisciplinary teams.

**Objective:**

Thus, the aim of this study was to examine to which extent it was possible to conduct interdisciplinary MRs at a psychiatric home and with patient involvement. The study was a mixed-method evaluation study that included 11 quantitative MRs analyzed by descriptive analysis and 5 qualitative semi-structured interviews analyzed by thematic coding analysis.

**Results:**

The MR performance formed the basis of an interview study. The patients' GPs and psychiatrist accepted 32 of the 37 (86 %) recommended changes to the current medication. At six months follow-up, 75 % of changes had been implemented. Three main interview themes and seven sub-themes were identified, covering collaboration between healthcare professionals, patient involvement, and the MR method. Conduction of interdisciplinary MRs was affected by e.g. challenges in the collaboration between GP and psychiatrists and patient involvement.

**Conclusion:**

Interdisciplinary MRs for psychiatric patients were seen as beneficial by healthcare professionals and had a high implementation rate of medication changes. In future use of the MR model, the involvement of patients and GPs should be ensured and include a psychiatrist in the MR team.

## 1 Introduction

Managing medications in primary care can be particularly challenging, especially for patients with psychiatric conditions. A scoping review identified non-adherence as a major drug-related issue, highlighting the need for targeted interventions to improve medication safety.^1^ Additionally, a qualitative study revealed that many healthcare professionals lack sufficient knowledge of psychotropic medications, leading to medication errors. Poor communication across care settings further exacerbates these issues.^2^ A multimethod study reinforced these findings, emphasizing the importance of improved communication, holistic patient support, and enhanced practitioner education to ensure safer medication management.^3^

In primary healthcare, medication reviews (MRs) have been seen to improve the appropriateness of somatic pharmacological treatment by, e.g. reducing drug-related problems.^4^, ^5^, ^6^, ^7^ Furthermore, studies have shown that somatic MRs prepared by an interdisciplinary team of several different healthcare professions may lead to an optimized MR process, reduced GP workload and increased medication safety.^8^, ^9^, ^10^, ^11^, ^12^, ^13^ Therefore, the authors now propose MRs in interdisciplinary teams for psychiatric patients.

In Denmark, many psychiatric patients and residents receive two or more antipsychotic drugs, i.e. antipsychotic polypharmacy, even though there is no evidence that antipsychotic polypharmacy results in an increased pharmacological effect.^14^^,^^15^ On the contrary, antipsychotic polypharmacy may increase the risk for side effects.^16^^,^^17^ Concomitant treatment with benzodiazepines and antipsychotic drugs only has a documented effect in the acute phase. A prolonged combination treatment increases the risk of adverse drug reactions, e.g., apathy, depression, and dementia. In Denmark, antipsychotic polypharmacy and combination treatment with antipsychotics and benzodiazepines are not recommended. In spite of this, the above mentioned treatments take place regularly, e.g., in a Danish psychiatric setting.^16^^,^^18^

Rational pharmacological treatment with antipsychotic drugs is a specialist task often handled by trained psychiatrists. However, in Denmark patients with psychiatric diseases, e.g. schizophrenia, who are in stable treatment may now be transferred from psychiatrist care to GP care with ongoing long-term antipsychotic treatment.^19^ This presents new responsibility challenges for general practitioners (GPs) who may feel that they are not sufficiently trained in prescribing and tapering psychiatric drugs.

Hence, some specific aspects should be considered for psychiatric patients receiving antipsychotic medication when conducting medication reviews, which are not seen amongst somatic patients, e.g.,

• Impaired cognitive capabilities of patients^20^, ^21^, ^22^, ^23^
• Possible drug addiction^24^^,^^25^
• Compulsory treatment order
• Challenges in the collaboration between GP and psychiatrist^23^
• GPs lack of knowledge on mental illness and medication^23^
• General shortage of psychiatrists^26^^,^^27^

Patient involvement has gained a growing focus in psychiatry. It might be even more critical in psychiatry because the patient's history and symptoms are crucial in diagnosing and evaluating medication.^28^ It has been described that both psychiatric patients and healthcare professionals in the psychiatric field prefer shared decision-making.^29^ Nevertheless, psychiatric patients feel that they are being overheard and don't have a say in their medication.^30^ MRs should be patient-centered, and it is ideal to include the patients' preferences for and experiences with medication to satisfy their needs and ensure adherence. Even though some reviews of the medication of psychiatric patients have been reported before,^31^, ^32^, ^33^, ^34^ we haven't been able to find any published studies about multidisciplinary MRs of psychiatric patients.

Thus, the aim of this study was to examine the possibility and benefit of conducting interdisciplinary MRs at a psychiatric home in collaboration between healthcare professionals and with patient involvement. Here, a special focus was on the above-mentioned specific aspects of the psychiatric setting.

## 2 Method

### 2.1 Setting and study design

This mixed-method evaluation study was a collaboration between the Medicine Unit at the Department of Clinical Pharmacology at Copenhagen University Hospital Bispebjerg and The Social Enterprise, responsible for several social psychiatric institutions in The Capital Region of Denmark. The social psychiatric home of this study was a residence and rehabilitation facility for adult patients with mental health problems with room for 30 residents.

This study consisted of two parts – a practical (1) and a study (2) part:

1. Practical medication review performance: The MRs of the psychiatric patients were performed at the psychiatric home by an interdisciplinary team consisting of the patients' medical doctors (MD), i.e., GP and psychiatrist, and caregivers, the individual patients, and a Medicine Team from the Medicine Unit (Table 1). The results of the MRs with a particular focus on the psychiatric treatment, i.e., recommended, accepted, and implemented medication changes, will only briefly be described in this paper. However, this part was a prerequisite for the following part as it formed the basis of part 2.

**Table 1:** Overview of study participants.

Participants	Role	Description	Included in part 1	Included in part 2
Residents	Patient	Adults with mental diagnoses unable to live by themselves. Schizophrenia was the most frequent diagnosis. Three residents had a compulsory treatment order (court-ordered to receive extended-release injectable antipsychotics). Three residents had an addiction.	Preferable	No
General Practitioner (GP)	Prescriber and responsible for somatic drugs	Oversaw prescription, tapering and management of somatic drugs.	Preferable	Yes
Psychiatrist	Prescriber and responsible for psychiatric drugs	Oversaw prescription, tapering and management of psychiatric drugs.	Preferable	Yes
Nurse	Medication history of the patient	Oversaw most of the communication to the patients' MDs on behalf of the patients.	Yes	Yes
Contact Person	Support and represent patient	Social- and health nursing assistants [social- og sundhedsassistent] or support workers with educational therapist training [pædagog] or non-health-related university degrees.	Yes	Yes
Medicine Team - Pharmacist	Consultant, MR lead	Clinical pharmacist (DAD)	Yes	No
Medicine Team – Consulting general practitioner	Consultant, MR lead	The consulting GP had a private practice and was not a GP for any patients in this project.	Yes	No
2. Qualitative study performance: The participants of the interdisciplinary MR team underwent semi-structured interviews about the MR method, the nature and extent of group collaboration, who had the responsibility for treatment, the degree of involvement of patients, and possible improvements to how MRs were conducted. Unfortunately, it became clear during the study design phase that the nurses at the psychiatric home did not believe that the residents could participate in the interview study due to their health condition.

An overview of study participants, their roles, and the part in which they participated in the study is presented below (Table 1).

### 2.2 Part 1: Practical medication review performance

**Fig. 1:**
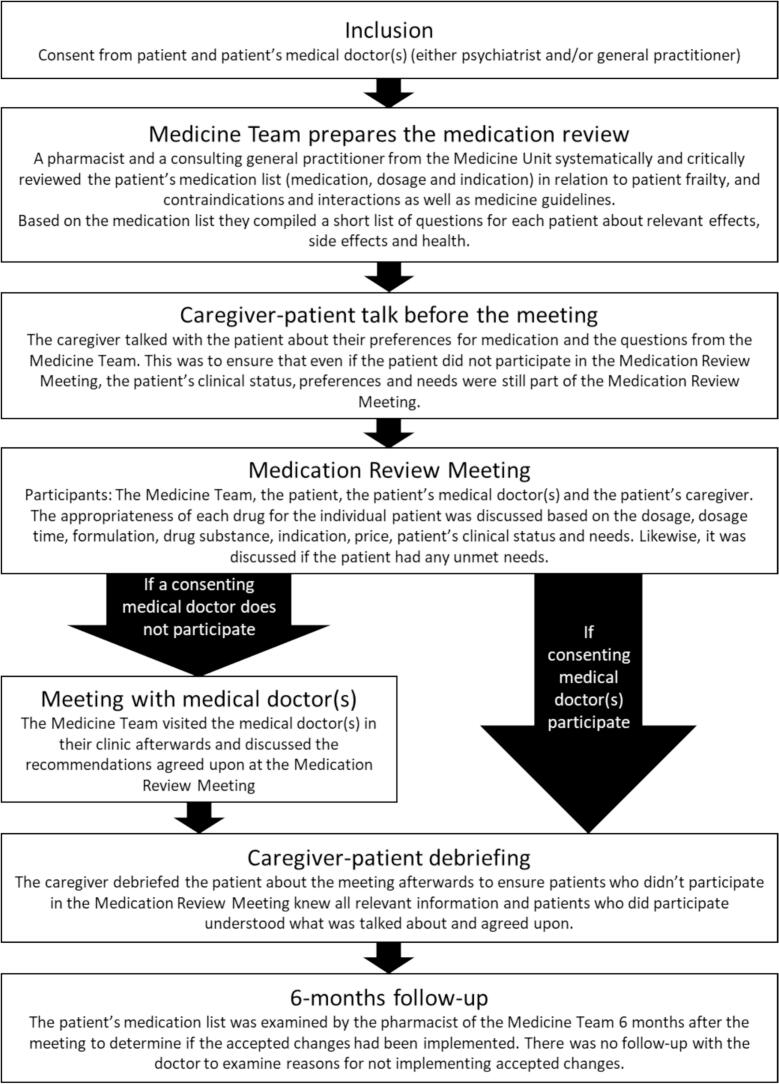
The process of the practical medication review.

Inspired by the authors' previous work on medication reviews,^8^ the practical MR was done in different steps involving different people; see Fig. 1 for the process.

All patients living at the psychiatric home, were invited to the project. Patients without any medication were excluded. Preferably, the patients participated in the “Medication Review Meetings”. However, this was not a requirement. The patient's MDs were invited to participate in the project, and at least one of them should consent to participate in the project for the patient to be included.

Even though the patients' MDs consented to participate in the project, they could object to participate in the “Medication Review Meeting”, e.g., due to time pressure. In these cases, the Medicine Team visited this MD in their clinic afterwards, i.e., at the “Meeting with medical doctor(s)” to discuss the recommended medication changes agreed upon at the “Medication Review Meeting” step in Fig. 1.

To ensure the patient's preferences and needs were considered during the MR, the contact person talked to the patient before the “Medication Review Meeting” (i.e., the step “Caregiver-patient talk before the meeting”, Fig. 1). The contact person asked both broad questions about the patient's preferences for the medication and questions about specific effects or side effects of specific medications of the patients' medication list. To ensure that all patients knew and understood what had been discussed at the medication review meetings, they all received a debriefing by caregivers after each meeting (Fig. 1).

### 2.3 Data collection

The Medication Review Team collected data about the patients' diagnoses, medical history and prescriptions from the residents' record at the psychiatric home in the period of March 2019 – January 2020. Recommended changes by the MR team and implemented changes by the doctors were registered. There was a particular focus on antipsychotic polypharmacy and simultaneously treatment with benzodiazepines and antipsychotics.^16^^,^^17^

### 2.4 Data analysis

A descriptive analysis was conducted to assess tendencies of changes in the number of patients across different medication categories before and after a medication review. All data was stored in the REDCap (Research Electronic Data Capture) tool,^35^^,^^36^ a secure database approved for data storage by the Danish Data Protection Agency. Data analysis was also carried out to some extent in REDCap. The rest of the data management and analysis was done using SAS® software (version 7.15 HF7 (7.100.5.6177) of SAS system Enterprise Guide. Copyright © 2017 SAS Institute Inc. Figures are made using Microsoft® Excel® software for Office 365 MSO (16.0.11328.20492).

### 2.5 Part 2: Qualitative study performance

Qualitative semi-structured interviews were used to explore the attitudes and potential benefit of the MR intervention with a special focus on communication, collaboration, and interactions between participants of the interdisciplinary team. Five interviews were conducted with GP, psychiatrist, nurse, and contact person. by an external anthropologist (LSD) who was not a part of the MR intervention. An interview guide was prepared focusing on psychiatric issues identified in study part 1. The interviewer explored the informants' previous experiences with interdisciplinary collaboration, their experiences participating in the MR project, their attitude towards implementing the interdisciplinary MR method in daily practice, and their views of potential areas for improvement. All interview guides were adapted to each informant's role in the interdisciplinary team. All interviews were audio recorded and transcribed verbatim.

Thematic coding analysis was used to analyze the interview data.^37^ Themes and subthemes were derived directly from the transcripts. Two researchers (SSH, DAD) independently coded and analyzed the data to ensure consistency and reliability. Through the coding process, when a disagreement over suitable themes corresponding to specific codes arose, any discrepancies were resolved through discussion between the two researchers until a consensus was reached. The interviews were transcribed using Express Scribe Transcription software.

## 3 Results

### 3.1 Part 1: Practical medication review performance

#### 3.1.1 Patient inclusion

**Fig. 2:**
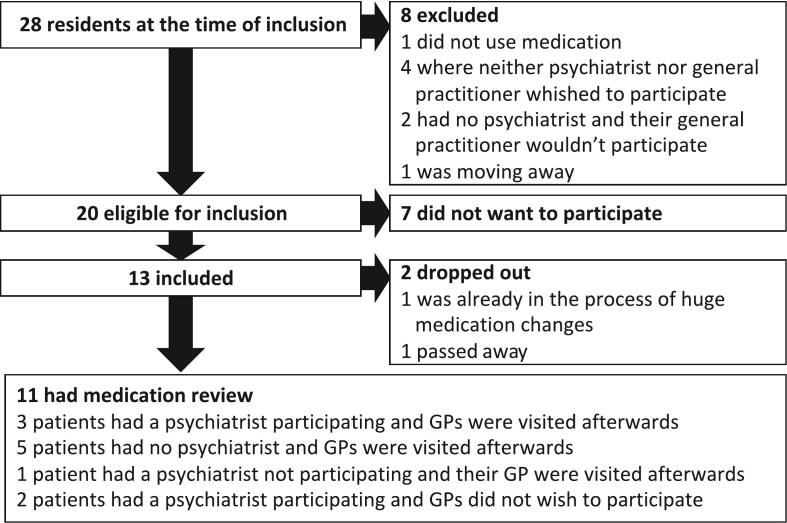
The flow of inclusion of the medication review.

Eleven patients (mean age 62 years) participated in this project, of which 7 (63 %) were female. See Fig. 2 for inclusion flow.

Patients had the following psychiatric diagnoses in the care record at the psychiatric home: Schizophrenia (catatonic, paranoid), (organic) personality disorder, (generalized) anxiety, depression, epilepsy, and skizo affective disorder. Three patients had known illegal drug abuse. Three patients had a compulsory treatment order.

#### 3.1.2 Medication review

To identify potential opportunities and barriers of the medication review method, we conducted MR Meetings for all 11 patients together with the caregivers. The GPs participated in none of the MR Meetings but preferred to be visited afterwards (See “Meeting with the medical doctor(s)” in Fig. 1). One patient tried to participate in the MR meeting but had to leave after a few minutes due to discomfort. One patient participated successfully in the “Meeting with the medical doctor(s)” with the GP.

We reviewed 102 prescriptions (mean 10 per patient), of which 30 % were as needed (see Table 2). The medical doctors accepted 32 of the 37 (86 %) recommended changes to current medication, including somatic and psychiatric medicines. Four of the five recommendations not accepted were discontinuations of somatic medication; half were as-needed prescriptions. The last recommendation not accepted was the discontinuation of a benzodiazepine. At six months follow-up, 75 % of accepted recommended changes had been implemented in full (66 %) or to some degree (9 %).

**Table 2:** Overview of prescriptions before medication review and the recommended, accepted and implemented changes of the medication review.

Class of medication	Prescriptions		Changes to prescriptions		
	Regular	As needed	Recommended	Accepted	Implemented or partly implemented
Total	71	31	37	32	24
Antipsychotics	15	3	9	9	6
Benzodiazepines	4	4	5	4	3
Antidepressants	3	0	1	1	1
Antiepileptics	2	0	0	–	–
Other psycho- active drugs	2	1	1	1	0
Opioids	5	1	3	3	2
Other somatic medication	40	22	18	14	12

At the “Medication Review Meeting”, three of four patients treated with a combination of antipsychotic drugs and benzodiazepines were recommended to discontinue their benzodiazepine. None of these patients had a psychiatrist, and the discontinuation of their benzodiazepine was therefore discussed with their GP. Two GPs accepted the discontinuation, but only one benzodiazepine was discontinued. At follow-up, 10 out of 11 patients received antipsychotic treatment, 3 out of 4 patients had concurrent use of regular antipsychotics and benzodiazepines and 1 out of 3 patients received more than one regular antipsychotic drug.

We recommended increasing the dose of antipsychotic medication for one patient, as the caregivers reported the patient had isolated themselves after a recent dose reduction.

Overall, the results of conducting interdisciplinary medication reviews for psychiatric patients indicate trends that the present medication review model could contribute to ensuring patients in psychiatric home receive more appropriate treatment in terms of both somatic and psychiatric medicine.

### 3.2 Part 2: Qualitative study performance

Five semi-structured interviews were conducted with two GPs, a psychiatrist, the psychiatric home nurse and one contact person (see Table 1) to elucidate the possibility and benefit of an interdisciplinary medication review collaboration for and with psychiatric patients.

The interviews were done the year after the medication review and lasted between 14 and 53 min (median 20 min). Two main themes and five subthemes were derived from the interviews (Table 3).

**Table 3:** Overview of interview themes, subthemes, and examples of quotes.

Theme	Subtheme	Quote
Collaboration between healthcare professionals	Treatment responsibility	*“So normally there is like a sharp line dividing who is responsible for what.” (GP1)* *“Their [the patients] medicine hasn't been looked at for years […] and no one really wants to look at it.” (nurse)*
	The healthcare profession's contributions and roles	*“They [the Medicine Team] did some of the work for me that I really should have done, but that I haven't really had time for.” (GP2)* *“We prescribe the medicine, but we don't always have control over how often they [the patient] take it. And there they [the nurse and the contact person] were good at saying: ‘Well, it's actually really difficult.’ Or: ‘It's only every other day.’ So, it gave a different insight.” (psychiatrist)*
Patient involvement	The present degree of patient involvement	*“There are some [patients] who like to manage it [the medicine] themselves and have some responsibility for it themselves, and that is good, but we like to be on the sideline because we have a responsibility when they live here.” (Nurse)* *“Many of the people I have at the residential care facility have such severe mental illness that we don't have long conversations.” (psychiatrist)*
	Suitability of patient involvement in the “Medication Review Meeting”	*“Yes, my resident has something to say. I think the other residents do too. You just have to work with them a little maybe.” (contact person)* *“It [involving the patient in the medication review] will take even longer.” (GP2)*
	Preference for better patient involvement	*“It can seem a bit discouraging [for the patient] if there are too many people. So, it may be better if you only have one person in there who listens to what is said and then can pass that on when they return to the resident.” (contact person)*

All interviewees agreed that carrying out interdisciplinary MRs was a good idea. The MRs enhanced professional sparring, allowed the staff to raise issues with the psychiatrist or the GP, provided new helpful knowledge about medicines use, helped the GPs, and provided a more thorough, critical, and comprehensive picture of the patients and their medication. Hence, the MRs ensured the most appropriate pharmacological treatment for the patient's benefit. The interviewees pointed out that each participant of the interdisciplinary team had a specific role and contributed something unique. The contact person said the primary motivators for the patient to participate in the project were the possibility of cheaper medication and fewer side effects.

#### 3.2.1 Collaboration between healthcare professionals

All interviewees pointed out collaboration as essential in securing the positive effect of interdisciplinary MRs. The interviewees agreed that the collaboration concerning the patients' medication was generally good. However, the interviewees' opinions on collaboration between healthcare professionals were divided into two subthemes: treatment responsibility and the healthcare profession's contributions and roles.

#### 3.2.2 Treatment responsibility

Several interviewees highlighted unclear treatment responsibility as a factor influencing the success of the interdisciplinary MR. The nurse felt a great responsibility for her patients but did not think it was her legal responsibility to oversee the medication. She problematized the fact that no one took responsibility for the patients' overall medication. The GPs expressed they lacked the skills to manage the psychiatric medication and were frustrated that the psychiatrist transitioned patient's care to them when patients were stabilized on antipsychotic medication. The nurse acknowledged that the staff at the residential facility had the relationship with the patients and that it could be difficult for the GPs to have an in-depth knowledge of the patients.

#### 3.2.3 The healthcare profession's contributions and roles

The interviewees pointed out that each interdisciplinary team member had a specific role and contributed something unique. The Medicine Team was particularly highlighted as beneficial for conducting interdisciplinary MRs as they freed up GP resources, functioned as an objective second opinion, advised on drug interactions, and had extensive knowledge of the different pharmaceutical preparations. The contact person and the nurse were also emphasized as advantageous as they had extensive knowledge about the patients' medication use, preferences, challenges, and medical history.

Both the nurse and the contact person believed that it was the GP's responsibility to implement the agreed upon changes, but the GP's implementation had been lacking. The contact person believed the deficient implementation may be due to the GP's forgetfulness. The nurse felt that the GPs should have implemented more accepted medicine changes as they were financially compensated for project participation. GP2 did not think he had been sufficiently compensated for his participation in the MRs, as it required a conversation with the patient and communication with the caregiving staff. However, he did not believe the lack of financial compensation was the reason why many GPs did not participate in the project, but rather a time pressure in general practice.

The nurse felt that the psychiatrist was more receptive to the recommended medication changes than the GP was, and she reasoned it was due to the psychiatrist being present at the “Medication Review Meeting”. The psychiatrist would also have preferred the GP to be present at the “Medication Review Meeting” to improve future collaboration and for the GP to make the final decisions on the patients' somatic medicine.

#### 3.2.4 Patient involvement

Suitability and perceived appropriateness of patient involvement in the interdisciplinary MRs were topics that all interviewees were asked about. The interviewees' opinions on patient involvement were divided into three subthemes: The present degree of patient involvement, suitability of patient involvement in the “Medication Review Meeting”; preference for better patient involvement.

#### 3.2.5 The present degree of patient involvement

The interviewees explained that the patients typically were involved in the everyday decisions about their medical treatment to varying degrees. Contact between healthcare professional and patient was made through the caregiving staff. Consequently, the caregiving staff assessed when a problem was sufficiently relevant to contact the GP for e.g., change in medication or a visit. The medicine was most often changed based on observations from the caregiving staff and possibly the GP, and not on information directly from the patient.

The psychiatrist said she visited the psychiatric home every 1–2 months to see her patients face to face. However, she was sometimes turned away by the patients as they did not want a conversation with her perhaps due to many of them having compulsory treatment orders and thereby treatment beyond their influence. This made patient involvement difficult.

GP1 experienced that the patient's annual chronic disease follow-up was forgotten, which was normally the time when the GP saw the patient directly.

#### 3.2.6 Suitability of patient involvement in the “Medication Review Meeting”

The nurse and the psychiatrist both believed that some of the patients would benefit from participating in the “Medication Review Meeting”. At the same time other patients would be too psychologically affected, e.g., get confused and afraid of side effects or the nature of the disease. GP2 did not think it would make sense to involve the patients in the “Medication Review Meeting” as it would simply make the meeting even more time-consuming.

The psychiatrist stated that her patients would not be able to participate as they were too mentally ill to be able to have long conversations about their medication. GP1 believed the patients were generally uninterested in participating in the “Medication Review Meeting”.

The contact person disagreed with GP1 and the psychiatrist as she experienced that her patient could be present during the entire”Meeting with the medical doctor(s)”. However, the contact person admitted that motivating the patients to go to the health services could sometimes be difficult. GP2 believed that the time consumption would increase by involving the patients.

#### 3.2.7 Preference for better patient involvement

Most of the interviewees agreed that patient involvement was important, and several expressed a preference for a higher degree of patient involvement.

GP1 had a desire to generally involve the patients more by asking about wishes and preferences during decision-making processes concerning medication. GP1 thought it was important that the patients were present when decisions were made about their medication unless the patients were too affected by their mental illness that participation would be harmful to them.

The nurse said that many of the patients at the psychiatric home, were not used to shared decision-making or to asking questions, as they had been living in institutions most of their lives and were used to someone deciding over them. The contact person suggested that the contact persons could make an extra effort to explain the project to the patients to increase the likelihood of patient participation and involvement. The contact person pointed out that the invitation to the project had to be understandable for the patients and should emphasize the possibility of saving money and getting less medicine as positive outcomes of participating in the project. However, the contact person emphasized that it could be overwhelming for the patient to attend the “Medication Review Meeting”. The contact person suggested that the patient only needed to attend a part of the “Medication Review Meeting”, e.g., discussing their needs and preferences at the start and then leave.

The contact person said that even though her patient participated in the “Meeting with medical doctor(s)”, they were not sufficiently questioned about their preferences and attitudes towards medication.

#### 3.2.8 Improvement of the Medication Review model

In addition to the two main themes, a theme about improving the MR model emerged. The interviewees also highlighted different suggestions for improvements, which could improve the possibility of conducting the interdisciplinary MR. Some suggestions for improvement were doing the interdisciplinary MRs as part of the annual chronic disease follow-up, participating in the “Medication Review Meeting” by video link, securing implementation of recommended medication changes directly at the “Medication Review Meeting”, and only involving the GP and the psychiatrist later in the MR process, for example after an initial screening of drug-related problems carried out by a pharmacist and/or nurse.

## 4 Discussion

This study examined the possibility and benefit of conducting interdisciplinary MRs at a psychiatric home in collaboration between healthcare professionals and with patient involvement. It was found that MR conduction was influenced by challenges in collaboration between the MDs, the GPs' lack of knowledge on mental illness and medication, and the involvement of patients due to e.g., the patient's mental state. These findings, though in other settings, correlated with the literature.^20^, ^21^, ^22^, ^23^, ^24^, ^25^ The patients included in the present study might differ from psychiatric patients in other studies, as they were not routinely being followed by specialist MDs at hospitals or outpatient clinics but also not living entirely independently.

It was expected that MRs of the psychiatric patients would be complicated by possible drug addiction and compulsory treatment order, e.g. compliance problems or high medication doses, based on findings in the literature.^24^^,^^25^ Drug addiction was not observed as a barrier in the MR intervention. However, a compulsory treatment order might influence the degree of patient involvement, which is discussed in more detail below.

In Denmark, it is recommended to avoid antipsychotic polypharmacy.^16^^,^^17^ In this study, we reduced the number of patients with antipsychotic polypharmacy by 66 %, which indicated unnecessary antipsychotic medication use. We recommended discontinuing benzodiazepines for three patients, all of whom didn't have a psychiatrist. The GPs were reluctant to this change, and only one discontinuation of benzodiazepines was implemented at follow-up. This may be caused by GPs not feeling they have sufficient experience and knowledge to handle and control psychiatric medicine, which was supported by the literature.^23^

### 4.1 Treatment responsibility

From the interviews it was clear the treatment responsibility was not sufficiently defined, which influenced the successful conduction of the interdisciplinary MR. Neither the psychiatrist nor the GPs wanted to take responsibility for the others' prescriptions. This was especially a problem for the patients who didn't have a psychiatrist or for those where their GP never visited them. GPs only visited patients if the staff requested it, so new somatic diseases or the development of known somatic diseases might get overlooked. Patients without psychiatrists might never get adjustments to their psychiatric medication, even when recommended by other professionals, as in this study. It could be argued that GPs could refer patients to a psychiatrist when they thought it was necessary to reevaluate and/or change the patients' psychiatric medication. However, the GPs might not feel they have the necessary knowledge and experience to assess when this time is. In addition, there is a shortage of psychiatrists in Denmark,^27^ which will make it difficult for GPs to transfer their psychiatric patients for treatment in psychiatry within a desired period.

Regular MRs could mitigate some of this and could be improved even more by including a consulting psychiatrist for those patients without a psychiatrist. Thereby the GPs wouldn't be responsible for changing psychiatric medication.

### 4.2 Patient involvement

The MRs were planned to include the patients, as the patients' preferences were assessed as important for the suitability and perceived appropriateness of the interdisciplinary MRs. However, in this project, we failed in involving the patients directly in the “Medication Review Meeting”. We did not examine why the patients didn't participate, as we were unable to include patients in the interview study. However, through interviews with the patients' MDs and the staff at the psychiatric home, we examined how the patients were generally involved in decisions about their treatment and why they might not participate. Many patients rarely initiated contact or communicated with their GPs, as the staff often did it on their behalf. Furthermore, the nurse told that even though they strived to make the patients able to deal with the healthcare system themselves, many patients were used to someone deciding over them. Half of the patients had compulsory treatment orders and thereby didn't have a say in their treatment, which could possibly also lead to a strained relationship with the psychiatrist. The patients' unfamiliarity with being included in decision-making about medication might be part of the reason why no patients participated. This is not uncommon, as another study has shown schizophrenia patients were less motivated to participate in medical decisions if they had a lower self-perceived decision-making skill.^38^

Most participants in the interview study agreed that patient involvement was essential in accordance with previous literature on both providers and patients with severe mental illness.^28^ GP1, the nurse and the psychiatrist all stated that the patients should be part of the “Medication Review Meeting” unless it would be psychological harmful. GP2, however, did not think it would make sense to include patients as it increases the time consumptions of the MR meetings. In the literature, some healthcare professionals agree upon this. They have reservations about this practice e.g., engaging patients in medication reviews requires additional time to explain the purpose and benefits, which can be challenging given the high workload of healthcare providers^39^ and patients can add complexity to the process, making it more time-consuming.^39^ Addressing these concerns involves improving communication strategies, providing patient education, and developing tools to facilitate patient engagement in medication reviews.^40^

The one patient who did try to participate in the “Medication Review Meeting” ended up leaving the meeting early. The patient's participation did seemingly not benefit either the patients or the performance of the MR intervention. This might indicate that at least some of these patients were unable to be included in the “Medication Review Meeting” and other ways to ensure patient involvement should be examined in the future.

Hence, in this project, the contact persons ended up representing the patients based on their knowledge of the patients as well as the “Caregiver-patient talk before meeting” (Fig. 1). The “Caregiver-patient talk before meeting” could be carried out in a more standardized way. A UK study used a one-page form on antipsychotics to increase patient involvement. Still, it also led to patients being alarmed by side effects, which was something the nurse also saw as a possible risk with patient involvement in the “Medication Review Meeting”.^41^ Consequently, patient information could be a relevant way to enhance patient involvement, but the use should be considered thoroughly so it is done in a way that is helpful for the patients and does not cause harm.

In future projects, it may be beneficial to have different approaches with different degrees of patient involvement which the patient and staff could choose from for each patient. These could range from the contact person representing the patient at the “Medication Review Meeting”, the patient participating in part(s) of the “Medication Review Meeting”, and the patient being present throughout the whole “Medication Review Meeting”.

### 4.3 The medication review method

In our study, the MDs accepted 86 % of recommended changes, which was relatively high when compared with existing literature on MRs for psychiatric patients (34 %, 76 %, and 93 %, respectively).^31^^,^^32^^,^^34^ The high acceptance rate indicates relevant recommendations and the interviews further supported this.

This interdisciplinary MR method involved many different healthcare professionals, which might be laborious. Still, in the interview, it was clear that each participant contributed with important and unique knowledge, which together made a thorough and holistic picture of the patient and their pharmacological treatment. The psychiatrist experienced that the contact person and the nurse had extensive knowledge about the patients' medication use, challenges, and medical history, which could reassure the psychiatrist to make the recommended changes. The GPs had a lower implementation rate, and this may be due to their absence at the “Medication Review Meeting”.

The GPs highlighted their positive attitude to the interdisciplinary MRs, but they did not feel sufficiently financially compensated for their time spent on the MRs (even though MRs were already included their collective agreement). GP2 believed that the real barrier to GP participation was time pressure and not finances. The scarcity of resources in general practice is a widespread problem according to the literature [43–44]. The performance of MRs in interdisciplinary teams can reduce GP workload.^10^^,^^13^ In future studies, it is important to conduct medication reviews in interdisciplinary teams to reduce the workload for GPs and to ensure GP participation to ensure that medication changes are effectively implemented by continuously updating the patient medical records and scheduling follow-ups, adjusting doses, or responding to side effects in a timely manner.

### 4.4 Limitations

It must be emphasized that the present study is based on 11 psychiatric MRs and five interview responses collected in a Danish setting. It is not our intention to generalize from this study but rather to evaluate the conduction of interdisciplinary MRs and describe aspects that may be related to the specific psychiatric setting. Furthermore, the Medicine Team was not interviewed, so the results did not include their point of view. However, this was done purposefully as the Medicine Team was a part of the study conceptualization and thereby biased on the assessment of the MR method and may also bias the results. Unfortunately, it was not possible to include psychiatric patients in the interview study due to their health condition. This is one of the study's greatest weaknesses. Going forward, it will be necessary to have a better understanding of this patient group to address barriers to involvement.

## 5 Conclusion

In conclusion, the present study showed that healthcare professionals saw interdisciplinary MRs for psychiatric patients as beneficial, and the MR's had an overall high implementation rate of changes to the medication. The successful conduction of interdisciplinary MRs was affected by the degree of collaboration of the healthcare professionals and patient involvement. The present study confirmed findings from the literature that several specific aspects were related to the psychiatric setting, such as impaired cognitive capabilities of patients, challenges in the collaboration between GP and psychiatrist, GPs' lack of knowledge on mental illness and medication, and general lack of available psychiatrists.

Even though almost no direct patient involvement was seen, the present study showed that contact persons could be used as representatives. In the interviews, it was empathized that the healthcare professionals involved in the MR each had a unique role and knowledge, which contributed to the complete picture of the patient and a thorough MR, reassured the MDs of the recommendations, and mitigated many of the specific challenges for psychiatric patients concerning medication. To mitigate these challenges even further, we propose in future projects to include a psychiatrist consultant in the Medicine Team to recommend and implement recommendations for psychiatric medication in collaboration with the patients' GP for those patients without a psychiatrist. In addition, GP participation should be ensured throughout the MR intervention process. A great weakness of the study was that it was not possible to include patients in the MR process or in the interview study. Future studies should incorporate, e.g., flexible patient involvement options, standardized pre-meeting input, decision-making support, efficient communication strategies, and alternative engagement methods to ensure meaningful and practical patient participation.

## Ethics approval and consent to participate

This project was approved by the Danish Data Protection Agency (I-Suite no 05564). According to Danish law, approval from The Danish Council on Ethics was not required and cannot be obtained for this study, as we only recommended changes to the medication. The GP and psychiatrist decided which changes to accept and implement as part of their regular care for the patient. Each patient gave informed written consent to be enrolled in the project. The study was conducted in compliance with the Helsinki Declaration in its latest form, good clinical practice guidelines, and followed the rules for informed consent. Written informed consent was obtained from all patients involved in the study as well as all participating clinic staff and general practitioners, ensuring confidentiality of data and anonymity of participants. It was stated that participants could withdraw from the study at any time. The GPs were reimbursed for their time on the project through their own “Quality and continued education foundation”.

## Consent for publication

Not applicable.

## Availability of data and materials

The dataset supporting the conclusions of this article is included within the article.

## Funding

This research received no external funding.

## Author contributions

Conceptualization, D.A.D. and C.V.; methodology, D.A.D., S.S.H., and C.V.; software, D.A.D.; validation, D.A.D. and S.S.H.; formal analysis, D.A.D. and S.S.H.; investigation, D.A.D, and L.S.D.; resources, C.V.; data curation, D.A.D.; writing—original draft preparation, D.A.D.; writing—review and editing, D.A.D., S.S.H., L.S.D. and C.V.; supervision, C.V.; project administration, D.A.D. All authors have read and agreed to the published version of the manuscript.

## CRediT authorship contribution statement

**Dagmar Abelone Dalin:** Conceptualization, Data curation, Formal analysis, Investigation, Software, Validation, Writing – original draft, Writing – review & editing. **Sara Sommer Holst:** Formal analysis, Methodology, Validation, Writing – review & editing. **Lucif Søemosegaard Dalin:** Investigation, Writing – review & editing. **Charlotte Vermehren:** Conceptualization, Methodology, Resources, Supervision, Writing – review & editing.

## Declaration of competing interest

The authors declare no conflict of interest.

## Appendix A Supplementary data

Supplementary material 1 Image 1

Supplementary material 2 Image 2
