# Supplementary material for: Interdisciplinary medication reviews of psychiatric patients – A mixed method evaluation
Source: Explor Res Clin Soc Pharm. 2025 Mar 2;18:100584. doi: 10.1016/j.rcsop.2025.100584 (PMC11952806; doi:10.1016/j.rcsop.2025.100584)
Supplement: Supplementary file 1 — Supplementary material 1 [file mmc1.docx]

Supplementary 1.

| Class of  medication | Recommended change | | | | | |
| --- | --- | --- | --- | --- | --- | --- |
|  | Discontinue | Reduce dosage | Increase dosage | Change to another drug | Change  time of  administration | Reduce pill-burden (eg. 1x10 mg instead of 2x5 mg) |
| **Total** | 26 | 3 | 1 | 4 | 1 | 2 |
| Antipsychotics | 6 | 1 | 1 | 1 |  |  |
| Benzodiazepines | 4 |  |  |  | 1 |  |
| Antidepressants |  |  |  |  |  | 1 |
| Antiepileptics |  |  |  |  |  |  |
| Other psycho- active drugs | 1 |  |  |  |  |  |
| Opioids | 1 | 1 |  | 1 |  |  |
| Other somatic medication | 14 | 1 |  | 2 |  | 1 |
