# Supplementary material for: Interdisciplinary medication reviews of psychiatric patients – A mixed method evaluation
Source: Explor Res Clin Soc Pharm. 2025 Mar 2;18:100584. doi: 10.1016/j.rcsop.2025.100584 (PMC11952806; doi:10.1016/j.rcsop.2025.100584)
Supplement: Supplementary file 2 — Supplementary material 2 [file mmc2.docx]

**Supplementary 2. Interview Guide**

**The Network Meeting**

- What made you decide to take part in this project involving medication reviews in network groups?
- Have you previously participated in a network group meeting or a similar setting? If so, how was the experience? What aspects were helpful, and what could have been improved?

**Your Regular Interaction with Residents and Healthcare Professionals**

- Can you describe your role in relation to the residents’ medication?
- How do you collaborate with different professionals (e.g., contact persons, psychiatrists, general practitioners (GPs), nurses) regarding residents’ medication?
- Have you ever interacted with a pharmacist concerning residents' medication? If so, in what context?

**The Network Group Meeting**

- What aspects of the meeting worked well?
- What challenges did you encounter? Do you have any suggestions for improvement?
- How did the presence or absence of the following professionals affect the meeting’s outcome?
  - The GP
  - The psychiatrist
  - The nurse
  - The medical team
- Did the network group meeting provide you with new knowledge? If so, what did you learn, and how?
- How can we encourage residents to participate in these meetings?
- What impact do you think resident participation would have on the process?

**Looking Ahead**

- Would you be willing to participate in another medication review within a network group setting?
- If you were to take part again, is there anything you would do differently?
- What would be needed for medication reviews in network groups to become a regular part of the workflow?
  - Would integrating them regularly be beneficial?
  - Do you have any suggestions for organizing these meetings so that all relevant practitioners (psychiatrist, GP, etc.) can participate more often?
- Have you made any changes to procedures or workflows after attending the network group meeting?
- Has your collaboration with colleagues (GP, psychiatrist, nurse, contact person) changed since the meeting? If so, in what way?

**Specific Questions**

**For the Psychiatrist**

- What are your thoughts on the proposed changes discussed during the network group meetings?
- Did you find them relevant? Did you agree with them?

**For the Contact Person**

- Before the network group meeting, you were encouraged to speak with the resident about their medication. How did that conversation go?
- What is your role when a resident wants or receives changes to their medication?
- Did you feel prepared to represent the resident? Did you feel confident in doing so?

**For the General Practitioner (GP)**

*(Since the GP did not attend the network group meeting, the previous section’s questions were not asked.)*

- Why did you not attend the network group meeting? Were you aware from the beginning that you wouldn’t be able to participate?
- Would receiving a fee influence your willingness to attend network group meetings?
- What would encourage you to participate in a medication review within a network group setting?
- Is there a different way we could present the project to increase GP participation?
